# Biological and chemical investigation of *Allium cepa* L. response to selenium inorganic compounds

**DOI:** 10.1007/s00216-014-7742-7

**Published:** 2014-03-21

**Authors:** M. Michalska-Kacymirow, E. Kurek, A. Smolis, M. Wierzbicka, E. Bulska

**Affiliations:** 1 College of Inter-Faculty Individual Studies in Natural Science and Mathematics, University of Warsaw, Żwirki i Wigury 93, 02-089 Warsaw, Poland; 2 Faculty of Chemistry, Biological and Chemical Research Centre, University of Warsaw, Żwirki i Wigury 101, 02-089 Warsaw, Poland; 3 Faculty of Biology, University of Warsaw, Miecznikowa 1, 02-096 Warsaw, Poland

**Keywords:** Speciation of selenium, *Allium cepa*, Hydroponic cultivation, Se-methylselenocysteine, Se-methionine, *Allium* test

## Abstract

The aim of this study was to evaluate the biological and chemical response of *Allium cepa* L. exposed to inorganic selenium compounds. Besides the investigation of the total content of selenium as well as its chemical speciation, the *Allium* test was used to evaluate the growth of onion roots and mitotic activity in the roots’ meristem. The total content of selenium was determined by inductively coupled plasma mass spectrometry (ICP MS). High-performance liquid chromatography (HPLC), coupled to ICP MS, was used for the selenium chemical speciation. Results indicated that *A. cepa* plants are able to biotransform inorganic selenium compounds into their organic derivatives, e.g., Se-methylselenocysteine from the Se(IV) inorganic precursor. Although the differences in the biotransformation of selenium are due mainly to the oxidation state of selenium, the experiment has also shown a fine effect of counter ions (H^+^, Na^+^, NH_4_^+^) on the response of plants and on the specific metabolism of selenium.

**Figure:**
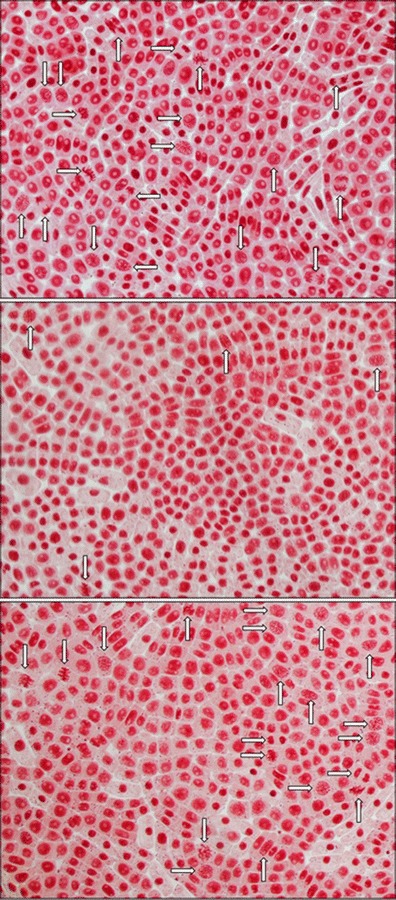
ᅟ

ᅟ

## Introduction

Selenium is an essential element for the proper functioning of humans and animals. Anticancer properties of selenium are of particular interest. Although the mechanisms of its activities are not fully understood, a clear correlation between the increased morbidity of cancer and the deficiencies of selenium in the diet was noted [1, 2]. Several investigations on populations from selenium-poor areas give statistically proven evidence that the selenium deficit increases cancer appearance, when compared to the areas rich in this element [3]. Therefore, all researches towards the development of dietary supplementation of selenium, which could compensate a natural deficiency of selenium in the human diet, are considered to be important.

It is expected that the consumption of vegetables, which have the ability to biotransform selenium inorganic forms into its organic derivatives—selenoaminoacids (e.g., Se-methylselenocysteine), which are most beneficial for humans, may play an important role in the prevention of cancer. Several studies were focused on vegetables from the *Allium* group, e.g., garlic, onion, and leek, which have the ability to take up large amounts of selenium [4–8]. Major selenium chemical species found in these selenized plants are Se-methylselenocysteine and its derivatives such as γ-glutamyl-Se-methylselenocysteine, which are known to be the most effective selenium inhibitors of tumor formation.

It was already found that the biotransformation of selenium in plants depends on the oxidation state of selenium in its inorganic precursors [4–6]. The aim of this study was to evaluate the specific biological response of *Allium cepa* L. plants grown hydroponically. Therefore, besides the investigation of the total content of selenium as well as its chemical speciation, the *Allium* test was used to evaluate the growth of onion roots and the mitotic activity in the roots’ meristem [8].

## Experimental

### Instrumentation

#### Inductively coupled plasma mass spectrometry

An ELAN 6100 DRC inductively coupled plasma mass spectrometer (ICP MS) (PerkinElmer SCIEX, USA) was used. A conventional Mainhardt nebulizer and a quartz cyclonic spray chamber were used for sample introduction. The ICP MS conditions were as follows: plasma power 1,100 W, plasma argon flow 15 L/min, auxiliary flow 1.21 L/min, and nebulizer flow 0.86 L/min. The interference-free isotope ^82^Se was monitored.

#### High-performance liquid chromatography

An Agilent 1200 (Agilent, USA) high-performance liquid chromatograph (HPLC) equipped with a Hamilton PRP-X100 anion exchange column, 250 mm × 4.6 mm i.d. and 10 μm (Hamilton, USA), was used in connection with ICP MS. The HPLC column was connected to the nebulizer of the ICP MS by a color-coded PEEK tubing (orange 1/16″, 0.020″; maximum pressure 5,000 psig; volume 100 μL).

### Chemicals and standards

All chemicals were of analytical grade and were used without further purification. Nitric acid (V) 65 %, hydrochloric acid 37 %, internal standard (yttrium), and multielemental ICP MS standard solutions were obtained from Merck (Merck, Germany). Water for the analysis was obtained from the Milli-Q System (Millipore, USA).

Sodium selenite (Na_2_SeO_3_), sodium hydrogen selenite (NaHSeO_3_), sodium selenate (Na_2_SeO_4_), and sodium-ammonium selenate (NaNH_4_SeO_4_) were obtained from Sigma, Germany. Selenomethionine (SeMet) and selenomethylselenocysteine (SeMetSeCys) were purchased from Sigma (USA). l-γ-Glutamyl-Se-methylseleno-l-cysteine (γ-glutamyl-SeMetSeCys) was obtained from PharmaSe Inc. (USA). Selenomethionine-Se-oxide (SeMetO) was achieved by adding an excess of oxidizing agent, 1 mL of 30 % (*v*/*v*) H_2_O_2_, to a 10-mL aliquot of a 0.1 mol/L HCI solution of selenomethionine (1 mg Se/L) and left overnight in the dark. Chemicals used for Knop’s medium, Ca(NO_3_), KNO_3_, KH_2_PO_4_, KCl, MgSO_4_, EDTA-Fe, were obtained from POCH (Poland).

The reference material BCR-402, white clover, with a certified total selenium content of 6.70 ± 0.25 μg/g, was obtained from the European Commission Joint Research Centre, Institute for Reference Materials and Measurements, Belgium.

### Plant cultivation

*A. cepa* L. plants were cultivated in 330-mL Erlenmeyer flasks at room temperature. After preincubation (24 h in distilled water, then 24 h in Knop’s medium), plants were transferred to Knop’s medium (control plants) or to Knop’s medium supplemented with selenium inorganic salts (test plants). The following selenium compounds were used: (i) anhydrous sodium selenite, (ii) sodium hydrogen selenite, and two Se(VI) salts: (iii) sodium selenate and (iv) sodium-ammonium selenate, all prepared up to the content of selenium of 10 mg/L. All solutions supplemented with selenium inorganic salts were replaced on a daily basis to assure a constant concentration of selenium during the cultivation period.

### Growth experiments

The length of roots and root growth rate (RGR) were inspected at the first day, than after 1, 2, 4, and 5 days of culture. The entire biomass was evaluated after 5 days of culture, by determining the fresh weight (FW) and dry weight (DW) of aerial and underground tissues. *Note*: for the DW, roots, leaves, and bulbs were dried at 30 °C for 72 h.

### *Allium* test

*Allium* tests were performed by cutting 2 mm of the root tips of each onion after 12, 24, 48, 72, and 96 h of cultivation; always, five root tips from each individual were used. They were macerated and stained in aceto-orcein (2 % orcein in 45 % acetic acid) for 24 h at room temperature. Two root tips were then squashed on each slide.

The mitotic activity in root tips was inspected under a light microscope, in the bright field. A mitotic index (number of dividing cells in 1,000 meristem cells) was counted for each slide.

### Chemical investigation

#### Determination of selenium

In order to determine the total content of selenium, approximately 0.1 g of plant tissue was digested in 8 mL of HNO_3_ (65 %) under the following conditions: (i) 500 W/10 min, (ii) 1,000 W/15 min, and (iii) cooling/5 min. A microwave-assisted unit (Anton Paar Multiwave Sample Preparation System, Austria) with Teflon vessels was used. Digested samples were filtered through sterile syringe-driven 0.45-μm nylon membrane filters (Millex, France). Transparent solutions were transferred to plastic tubes, filled with deionized water to 20 mL, and stored in a refrigerator below 4 °C before measurements.

Calibration was performed with the standard solutions containing 1, 10, and 100 μg/L of selenium, and yttrium (10 μg/L) was used as an internal standard for instrument drift correction. The total measurement time was 25 s.

The validation of the analytical procedure was performed with certified reference material BCR-402, white clover, with total selenium content of 6.70 ± 0.25 μg/g and which was digested in the same conditions as described above. The obtained result of 6.74 ± 0.68 μg/g was considered as being within the uncertainty of the certified value.

#### Extraction procedure

After 5 days of cultivation, all control and test plants were thoroughly cleaned under running tap water and subsequently rinsed with distilled water. Wet roots were weighted and then dried at 30 °C for 72 h. About 0.1 g of dry mass was weighted in the plastic centrifuge tubes; then, 1 mL of water was added. The extraction supported by ultrasonic shaking (30 °C for 30 min) was performed with water. The supernatant was then separated from the residue by centrifugation for 15 min at 13,000 rpm and filtered through 0.45-μm membrane filters. Aliquots used for HPLC separation were kept at −10 °C before measurements.

#### Chromatographic separation procedure

The conditions for HPLC chromatographic separation were as follows. Eluent A, 5 mM acetate buffer (pH 4.7), and eluent B, 150 mM acetate buffer (pH 4.7), were used. The mobile phase was delivered at 1.0 mL/min in gradient mode: 0–4 min—100 % A, 4–7 min—from 100 % A to 0 % A, and 7–30 min—100 % B. The conditions for HPLC chromatographic separation were optimized for the needs of the experiment. Filtered solutions were degassed before the injection; then, 100 μL of the sample was injected for HPLC ICP MS measurements.

The standard solutions were prepared to obtain the concentration of 500 μg/L of selenium. Retention times were SeMetO = 2.2 min, SeMetSeCys = 2.8 min, SeMet = 5.0 min, Se(IV) = 9.8 min, γ-glutamyl-SeMetSeCys = 13.0 min, and Se(VI) = 15.0 min.

## Results and discussion

### Plant growth

The growth of roots was examined visually for control and test plants within 5 days. The roots of plants cultivated in the presence of Se(IV) compounds (Na_2_SeO_3_ or NaHSeO_3_) were significantly shorter compared to those treated with Se(VI) compounds (Na_2_SeO_4_·10H_2_O or NaNH_4_SeO_4_). After 96 h, the average increment of onion roots cultivated in the presence of sodium selenite, hydrogen sodium selenite, sodium selenate, and sodium-ammonium selenate was, respectively, 2.0, 1.3, 4.1, and 4.2 cm. As it was expected, the highest growth rate was found for the control plants (6.2 cm).

Clearly, all inorganic compounds inhibit root growth, and the most pronounced inhibition effects were observed in the presence of Se(IV), with a less pronounced but still significant difference between sodium selenite and sodium hydrogen selenite. The presence of Se(VI) does not depend significantly on the type of counter ion.

The effect of selenium on the growth of roots was also supported by evaluating the amount of biomass (after 96 h). The weight of the fresh roots of plants exposed to Se(IV) or Se(VI) was 52 or 67 %, respectively, of that of the control plant.

### Mitotic activity

**Fig. 1:**
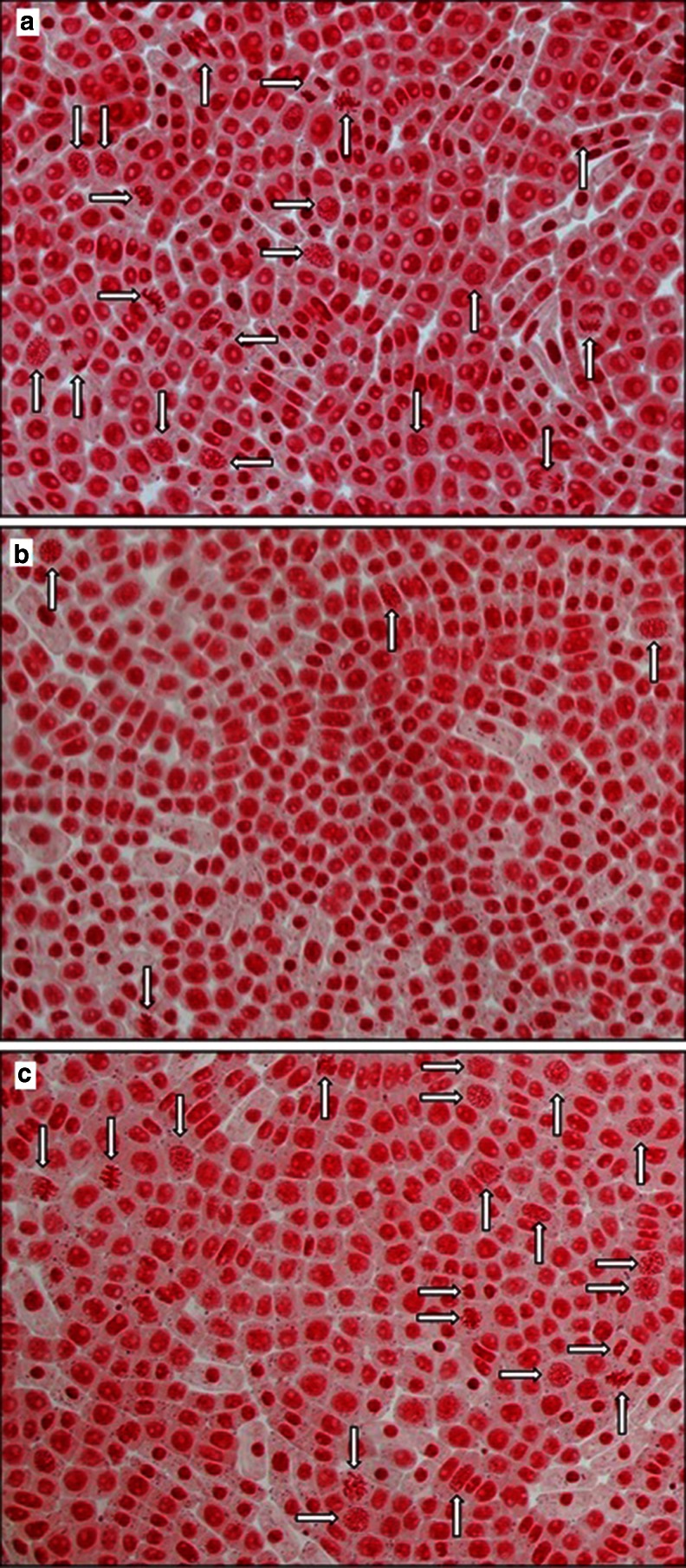
Mitotic divisions (*white arrows*) of meristematic plant cells in *Allium cepa* L. grown in Knop’s medium: **a** no additives, **b** with Se(IV), and **c** with Se(VI). Magnification ×400

**Fig. 2:**
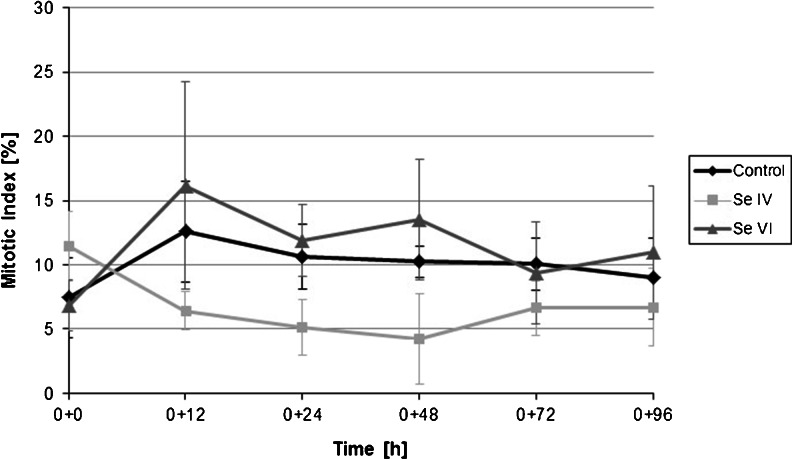
Mitotic index (expressed as a percentage of dividing cells) for root tips of *Allium cepa* L. *Diamonds* control plants; *squares and triangles* plants cultivated in Knop’s medium enriched with sodium selenite (IV) and sodium selenate (VI), respectively. The concentration of selenium was constantly 10 mg/L

The evaluation of the mitotic activity of root tips was performed for Na_2_SeO_3_ and Na_2_SeO_4_. The biological responses of the root cells are shown in Fig. 1a–c. Interestingly, the mitotic activity of root tips was not reduced in the presence of Na_2_SeO_4_ (Fig. 1a, c); however, the significant reduction of the number of divisions was observed in the presence of Se(IV) (Fig. 1b). Although both inorganic selenium compounds decreased the growth of roots, in the case of Na_2_SeO_3_, the inhibitory effect was expressed also in the mitotic divisions, resulting in a reduction of the mitotic index (Fig. 2).

### Uptake of selenium by roots

The total content of selenium in the onion roots depends on its chemical form. The results indicate that the process of its uptake varies between Se(IV) and Se(VI) compounds. The average content of selenium in roots grown in the presence of various inorganic selenium compounds is listed in Table 1. Although Se(IV) affects the plants significantly stronger than Se(VI) compounds, its uptake efficiency is almost three times lower. The uptake efficiency depends significantly on the selenium oxidation stage rate; moreover, it was also associated with the type of salt, specifically the counter ions, for both Se(IV) and Se(VI) species.

**Table 1:** The content of selenium in roots (dry mass) grown in the presence of selected selenium compounds

Compound	Total content^a^ [mg/kg]
Na_2_SeO_3_	670 ± 15
NaHSeO_3_	979 ± 15
Na_2_SeO_4_·10H_2_O	2,700 ± 25
NaNH_4_SeO_4_	2,100 ± 20

^a^The value ± expanded uncertainty (*k* = 2) for the measurements

### Selenium speciation

In order to evaluate selenium chemical speciation, roots of three individual plants from each of the test culture were collected. Thus, five sets of plants were examined: (i) control plants and those exposed to (ii) sodium selenite, (iii) sodium hydrogen selenite, (iv) sodium selenate, and (v) sodium-ammonium selenate. The following standard compounds were used for the calibration of the retention time of chromatographic separation: SeMetO (2.2 min), SeMetSeCys (2.8 min), SeMet (5.0 min), Se(IV) (9.8 min), γ-glutamyl-SeMetSeCys (13.0 min), and Se(VI) (15.0 min). On the basis of the retention time, the presence of those compounds in the extracts from plant tissues was identified.

**Fig. 3:**
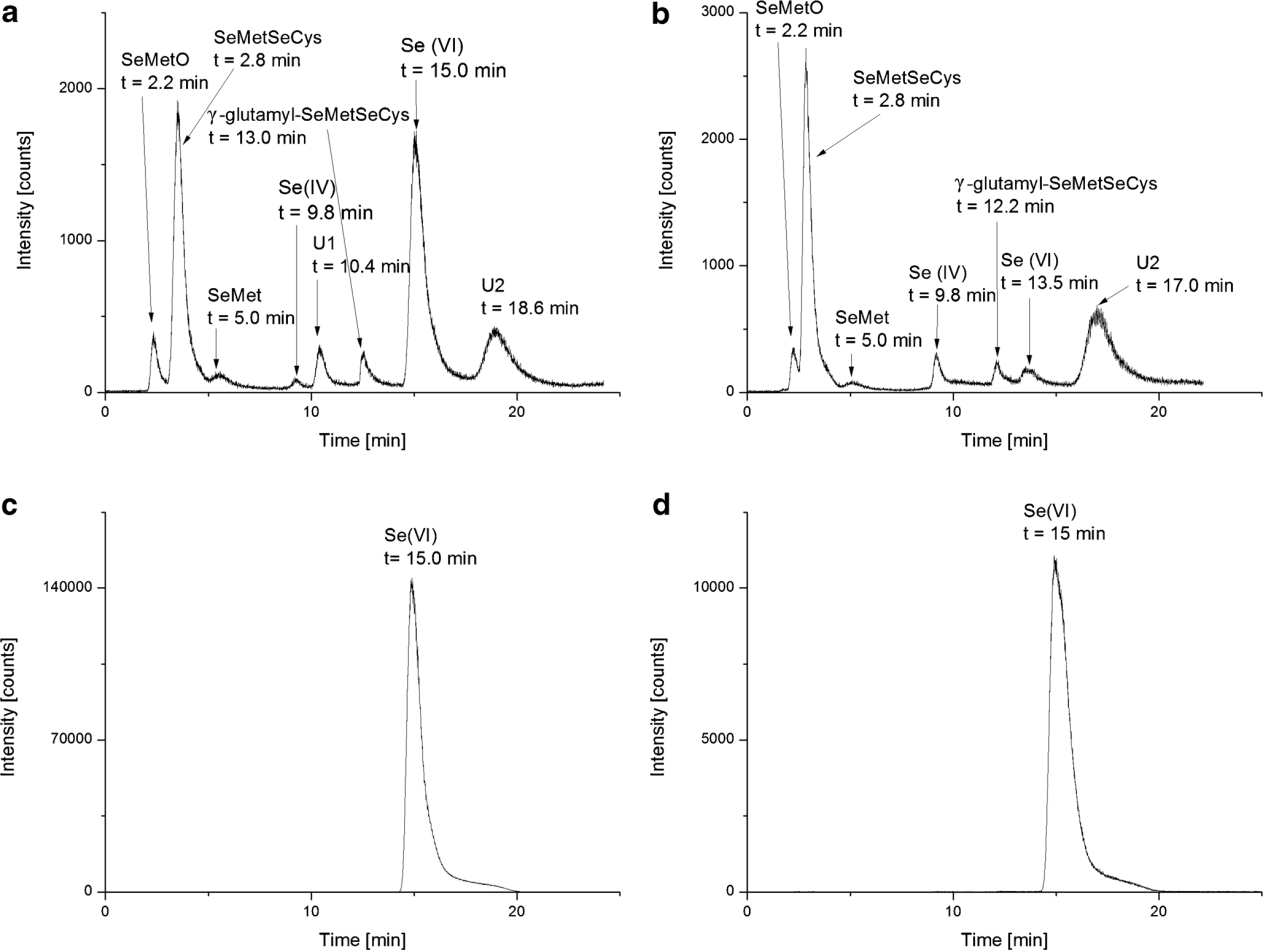
Chromatograms obtained from the extracts of the roots of plants grown in Knop’s medium enriched with **a** Na_2_SeO_3_, **b** NaHSeO_3_, **c** Na_2_SeO_4_, and **d** NaNH_4_SeO_4_

In plants treated with Se(VI), the presence of only one original form of selenium was revealed (Fig. 3c, d). In the case of plants treated with Se(IV), all above-listed selenium species (standard compounds) were identified. Two signals at *R*_t_ = 10.4 min and *R*_t_ = 18.6 min were not identified due to the lack of respective standard compounds (Fig. 3a, b). Thus, the ability of *A. cepa* L. plants to metabolize inorganic species of Se(IV) to its organic derivatives was confirmed [4, 5]. A similar effect of the metabolism of inorganic precursors of selenium was observed also for other plants, e.g., *Allium schoenoprasum* [6].

The detailed examination of the chromatograms, especially Fig. 3a, b, allows also the detection of the different responses due to the counter ions at Se(IV) compounds. The effect was most pronounced in the case of abundant species, SeMetSeCys. The content of selenium in the fractions of SeMetSeCys was 201 and 107 mg/kg when onion roots were grown in the presence of NaHSeO_3_ and Na_2_SeO_3_, respectively. This is in line with the data on the efficiency of uptake of selenium (Table 1). The contrary effect was noticed for Se(VI); the signal recorded at 15.0 min was more than two times higher for the plant exposed to sodium selenite compared to that for the plant exposed to sodium hydrogen selenite (16 mg/kg). These results indicate that the biotransformation of selenium depends not only on the oxidation state of selenium, but also on the counter ions. The presence of NaHSeO_3_ not only supports the efficiency of the uptake of selenium, but also promotes the biotransformation of the inorganic precursor to its organic derivatives, mainly SeMetSeCys.

## Conclusions

In this work, biological and chemical methods were applied in order to obtain complementary information on the plants’ (*A. cepa* L.) response to the different inorganic salts of selenium (NaSeO_3_, NaHSeO_3_, Na_2_SeO_4_, NaNH_4_SeO_4_). A significant influence of the oxidation state of selenium on its metabolism in plants as well as on the mitotic activity in the roots’ meristem has been confirmed. The use of the *Allium* test as well as observing the growth of the biomass of the onion roots enabled the evaluation of the response of *A. cepa* on the presence of various inorganic selenium compounds and the demonstration of the effect of counter ions (H^+^, Na^+^, NH_4_^+^), most pronounced in the case of Se(IV) compounds.
